# FEASIBILITY OF A PHYSICAL ACTIVITY GOAL SETTING INTERVENTION AMONG INSUFFICIENTLY ACTIVE MIDLIFE ADULTS

**DOI:** 10.1093/geroni/igae098.4192

**Published:** 2024-12-31

**Authors:** Molly Maxfield, Keenan Pituch, Dereck Salisbury, Fang Yu, Rodney Joseph

**Affiliations:** Arizona State University, Phoenix, Arizona, United States; Arizona State University, Phoenix, Arizona, United States; University of Minnesota, University of Minnesota, Minnesota, United States; Arizona State University, Phoenix, Arizona, United States; Arizona State University, Phoenix, Arizona, United States

## Abstract

Physical activity (PA) during midlife can reduce risk for Alzheimer’s disease and related dementias. However, adherence to national PA guidelines (≥150 min/week of moderate-to-vigorous intensity aerobic PA [MVPA]) among midlife adults is rarely achieved through behavioral PA interventions, which may be due to inconsistent or unsuitable approaches to goal setting. The purpose of this study was to assess the feasibility of a 6-month virtual coaching intervention designed to test the effectiveness of three different goal setting techniques to enhance mechanisms of self-regulation and self-efficacy and increase PA among insufficiently active midlife adults with obesity. Study participants (n=24) were provided a Fitbit to self-monitor their MVPA, received weekly virtual PA coaching conducted via Zoom, and were assigned to 1 of 4 groups: i) static weekly goal of 150 min/week of MVPA, ii) weekly self-selected MVPA goals, iii) modest incremental increase in weekly MVPA goal (20% min/week greater than previous week’s MVPA); or iv) non-goal setting comparison group. Results showed favorable outcomes for retention (91.3% [n=21] retained at 6-months), coaching sessions attendance (85.9% of sessions completed), and Fitbit wear adherence (Fitbit worn for > 10 hour/day on 87.1% of intervention days). Although the small sample precludes use of inferential statistics or examination of group differences, patterns of change suggested that participants experienced improvements in MVPA and psychosocial mediators of self-efficacy and self-regulation. Building upon these results, a forthcoming Stage 2 proof-of-concept trial (n=120) will be conducted to examine the effectiveness of these goal-setting techniques to increase MVPA among midlife adults.

